# Supplementary material for: The epidemiology, treatment patterns, healthcare utilizations and costs of Acute Myeloid Leukaemia (AML) in Taiwan
Source: PLoS One. 2022 Jan 21;17(1):e0261871. doi: 10.1371/journal.pone.0261871 (PMC8782483; doi:10.1371/journal.pone.0261871)
Supplement: S1 Fig — (DOCX) [file pone.0261871.s001.docx]

**S1 Figure.** Study design for the analysis of healthcare utilization and costs
