# Supplementary material for: The epidemiology, treatment patterns, healthcare utilizations and costs of Acute Myeloid Leukaemia (AML) in Taiwan
Source: PLoS One. 2022 Jan 21;17(1):e0261871. doi: 10.1371/journal.pone.0261871 (PMC8782483; doi:10.1371/journal.pone.0261871)
Supplement: S1 Table — (DOCX) [file pone.0261871.s002.docx]

## S1 Table. 2008 WHO Classification and ICD-O-3 codes for Acute Myeloid Leukaemia

| **Acute Myeloid Leukemia** | **2008 WHO Classification** | **ICD-O-3 codes** |
| --- | --- | --- |
| AML with recurrent genetic abnormalities | 98663/ 98713/ 98963/ 98973/ 98653/ 98693/ 99113 | 98663/ 98713/ 98963/ 98973 |
| AML with myelodysplasia-related changes | 98953 | 98953 |
| Therapy-related myeloid neoplasms | 99203 | 99203 |
| Acute myeloid leukemia, NOS | 98613 | 98613 |
| Other acute myeloid leukemia | 98403/ 98673/ 98703/ 98723/ 98733/ 98743/ 98913/ 99103/ 99313 | 98403/ 98673/ 98703 /98723/ 98733/ 98743/ 98913/ 99103/ 99313 |
| Myeloid sarcoma | 99303 | 99303 |
| Myeloid proliferations related to Down Syndrome | 98983 | - |
| Blastic plasmacytoid dendritic cell neoplasm | 97273 | 97273 |
