# Supplementary material for: The epidemiology, treatment patterns, healthcare utilizations and costs of Acute Myeloid Leukaemia (AML) in Taiwan
Source: PLoS One. 2022 Jan 21;17(1):e0261871. doi: 10.1371/journal.pone.0261871 (PMC8782483; doi:10.1371/journal.pone.0261871)
Supplement: S2 Table — (DOCX) [file pone.0261871.s003.docx]

**S2 Table**. 2006-2015 Annual incidence of acute myeloid leukaemia stratified by overall, gender and age.

|  |  |  | 2006 | 2007 | 2008 | 2009 | 2010 | 2011 | 2012 | 2013 | 2014 | 2015 | |
| --- | --- | --- | --- | --- | --- | --- | --- | --- | --- | --- | --- | --- | --- |
| **Total** |  |  |  |  |  |  |  |  |  |  |  |  | |
| Patient number | |  | 636 | 711 | 689 | 758 | 758 | 750 | 807 | 771 | 768 | 755 | |
| Crude rate^†^  ^*^Age-standardized rate^†^ | | | 2.78 | 3.01 | 2.99 | 3.28 | 3.27 | 3.23 | 3.46 | 3.30 | 3.28 | 3.21 | |
|  |  |  | 2.47 | 2.76 | 2.58 | 2.75 | 2.62 | 2.67 | 2.76 | 2.58 | 2.44 | 2.41 | |
| Median Age |  |  | 57 | 57 | 60 | 59 | 61 | 60 | 60 | 61 | 60 | 61 | |
| **Male** |  |  |  |  |  |  |  |  |  |  |  |  | |
| Patient number (N) | | | 374 | 426 | 370 | 446 | 437 | 437 | 478 | 441 | 426 | 411 | |
| Patient number (%) | | | 58.81 | 59.92 | 53.70 | 58.84 | 57.65 | 58.27 | 59.23 | 57.20 | 55.47 | 54.44 | |
| Crude rate^†^ |  |  | 3.23 | 3.67 | 3.18 | 3.83 | 3.76 | 3.75 | 4.09 | 3.77 | 3.64 | 3.51 |  |
| ^*^Age-standardized rate^†^ | | | 2.88 | 3.23 | 2.79 | 3.22 | 3.07 | 3.13 | 3.27 | 3.00 | 2.80 | 2.65 |  |
| Median Age |  |  | 59 | 62.5 | 62 | 61 | 62 | 60 | 61 | 62 | 61 | 63 | |
| **Female** |  |  |  |  |  |  |  |  |  |  |  |  | |
| Patient number (N) | | | 262 | 285 | 319 | 312 | 321 | 313 | 329 | 330 | 342 | 344 | |
| Patient number (%) | | | 41.19 | 40.08 | 46.30 | 41.16 | 42.35 | 41.73 | 40.77 | 42.80 | 44.53 | 45.56 | |
| Crude rate^†^ |  |  | 2.32 | 2.51 | 2.80 | 2.72 | 2.78 | 2.70 | 2.83 | 2.82 | 2.91 | 2.92 | |
| ^*^Age-standardized rate^†^ | | | 2.06 | 2.28 | 2.38 | 2.29 | 2.20 | 2.25 | 2.28 | 2.19 | 2.10 | 2.21 | |
| Median Age |  |  | 53 | 54 | 58 | 55 | 59 | 60 | 57 | 60 | 59.5 | 57 | |
| **Age [0-19 yrs]** | | |  |  |  |  |  |  |  |  |  |  | |
| Patient number (N) | | | 60 | 60 | 58 | 65 | 37 | 63 | 57 | 52 | 34 | 47 | |
| Crude rate† | | | 1.04 | 1.06 | 1.05 | 1.20 | 0.71 | 1.23 | 1.13 | 1.06 | 0.71 | 1.00 | |
| **Age [20-39 yrs]** | | |  |  |  |  |  |  |  |  |  |  | |
| Patient number (N) | | | 94 | 119 | 97 | 106 | 117 | 99 | 109 | 93 | 108 | 91 | |
| Crude rate† | | | 1.26 | 1.61 | 1.31 | 1.45 | 1.60 | 1.37 | 1.51 | 1.29 | 1.51 | 1.28 | |
| **Age [40-59 yrs]** | | |  |  |  |  |  |  |  |  |  |  | |
| Patient number (N) | | | 183 | 195 | 184 | 219 | 213 | 205 | 231 | 211 | 231 | 222 | |
| Crude rate† | | | 2.76 | 2.88 | 2.66 | 3.12 | 2.99 | 2.86 | 3.21 | 2.93 | 3.20 | 3.07 | |
| **Age [60+ yrs]** | | |  |  |  |  |  |  |  |  |  |  | |
| Patient number (N) | | | 299 | 337 | 350 | 368 | 391 | 383 | 410 | 415 | 395 | 395 | |
| Crude rate† | | | 9.79 | 10.76 | 10.81 | 10.93 | 11.12 | 10.34 | 10.55 | 10.20 | 9.28 | 8.86 | |
| **Sex ratio** | | | 1.39 | 1.46 | 1.14 | 1.41 | 1.35 | 1.39 | 1.45 | 1.34 | 1.25 | 1.2 | |

†Per 100,000 individuals; *According to WHO 2000 standardized population
