# Supplementary material for: The epidemiology, treatment patterns, healthcare utilizations and costs of Acute Myeloid Leukaemia (AML) in Taiwan
Source: PLoS One. 2022 Jan 21;17(1):e0261871. doi: 10.1371/journal.pone.0261871 (PMC8782483; doi:10.1371/journal.pone.0261871)
Supplement: S3 Table — (DOCX) [file pone.0261871.s004.docx]

**S3 Table.** **Healthcare utilization and costs of AML.** The first, second, and third years (since induction therapy) of healthcare utilization and costs of AML; stratified by induction therapy and stem cell transplantation (HSCT)

|  | | *The 1st year* | | *The 2nd year* | | *The 3rd year* | | *Until end of follow-up* | |
| --- | --- | --- | --- | --- | --- | --- | --- | --- | --- |
| **HDAC with HSCT** | | | | | | | | | |
| Patient no. | | 52 |  | 46 |  | 26 |  | 52 |  |
| Follow-up time, years | | | | | | | | | |
|  | Mean (SD) | 1.0 | (0.1) | 1.8 | (0.3) | 2.8 | (0.3) | 2.7 | (1.7) |
|  | Median (IQR) | 1.0 | (0) | 2.0 | (0.5 | 3.0 | (0.3) | 2.0 | (2.7) |
| *Medical cost | | | | | | | | | |
|  | Mean (SD) | 85715.8 | 34657.0 | 38630.6 | 42118.2 | 21031.2 | 22805.8 | 139155.9 | 64668.1 |
|  | Median (IQR) | 78876.2 | (46837.7) | 26832.4 | (47705.4) | 12139.3 | (36217.3) | 121021.8 | (69565.5) |
| †No. of OPD visits | | | | | | | | | |
|  | Mean (SD) | 31.6 | (16.0) | 34.5 | (20.5) | 41.7 | (23.6) | 108.3 | (97.4) |
|  | Median (IQR) | 27 | (20) | 28 | (35) | 40 | (44) | 75 | (110) |
| *Cost of OPD visits | | | | | | | | | |
|  | Mean (SD) | 6676.0 | 7059.2 | 6742.9 | 7790.4 | 5548.5 | 5701.6 | 18126.9 | 21028.9 |
|  | Median (IQR) | 4275.7 | (6853.4) | 5263.7 | (6593.4) | 4175.6 | (7110.7) | 12642.1 | (11691.8) |
| †No. of hospitalization | | | | | | | | | |
|  | Mean (SD) | 5.6 | (2.2) | 3.1 | (1.6) | 3.0 | (1.3) | 9.4 | (4.5) |
|  | Median (IQR) | 5 | (4-7) | 3 | (2-4) | 3 | (2-4) | 10 | (5-12) |
| ‡Length of stay, days | | | | | | | | | |
|  | Mean (SD) | 168.3 | (60.0) | 85.6 | (61.7) | 59.2 | (50.1) | 255.8 | (101.7) |
|  | Median (IQR) | 154 | (127-208) | 77 | (37-132) | 49 | (23-83) | 240 | (167-320) |
| *Cost of hospitalization | | | | | | | | | |
|  | Mean (SD) | 79039.8 | 33009.6 | 31887.7 | 41545.2 | 15482.7 | 20842.4 | 121028.9 | 54412.8 |
|  | Median (IQR) | 73833.5 | (57211.7-103513.3) | 15035.8 | (0-40913.6) | 2488.7 | (0-24176.6) | 108222.4 | (86878.3-142016.5) |
| **HDAC with chemotherapy alone** | | | | | | | | | |
| Patient no. | | 110 |  | 43 |  | 25 |  | 110 |  |
| Follow-up time, years | | | | | | | | | |
|  | Mean (SD) | 0.6 | (0.4) | 1.8 | (0.3) | 2.8 | (0.3) | 1.4 | (1.7) |
|  | Median (IQR) | 0.7 | (0.2-1.0) | 2.0 | (1.6-2.0) | 3.0 | (2.7-3.0) | 0.7 | (0.2-2.0) |
| *Medical cost | | | | | | | | | |
|  | Mean (SD) | 48125.5 | 30492.9 | 12083.7 | 16986.2 | 2778.0 | 6659.7 | 54472.7 | 36286.5 |
|  | Median (IQR) | 42271.2 | (26751.3-65812.9) | 2818.6 | (813.8-18097.7) | 881.0 | (213.5-1480.3) | 46529.4 | (27433.5-76617.5) |
| †No. of OPD visits | | | | | | | | | |
|  | Mean (SD) | 19.4 | (18.2) | 33.6 | (22.9) | 24.7 | (19.3) | 45.9 | (62.2) |
|  | Median (IQR) | 17 | (2-29) | 26 | (21-45) | 23 | (9-34) | 23 | (2-62) |
| *Cost of OPD visits | | | | | | | | | |
|  | Mean (SD) | 3138.6 | 4644.3 | 3376.6 | 5194.9 | 1725.5 | 2967.8 | 5112.3 | 7360.8 |
|  | Median (IQR) | 1323.8 | (340-3642.7) | 1178.9 | (442.7-3378.8) | 822.9 | (213.5-1473.5) | 1699.4 | (493.5-7095.7) |
| †No. of hospitalization | | | | | | | | | |
|  | Mean (SD) | 3.6 | (2.3) | 2.6 | (1.7) | 1.7 | (0.6) | 4.4 | (3.9) |
|  | Median (IQR) | 4 | (1-5) | 3 | (1-4) | 2 | (1-2) | 4 | (1-6) |
| ‡Length of stay, days | | | | | | | | | |
|  | Mean (SD) | 109.1 | (65.5) | 45.6 | (42.1) | 22.0 | (16.1) | 119.8 | (76.2) |
|  | Median (IQR) | 111 | (52-150) | 33 | (10-68) | 24 | (5-37) | 123 | (52-167) |
| *Cost of hospitalization | | | | | | | | | |
|  | Mean (SD) | 1499.6 | 968.0 | 290.2 | 469.9 | 35.1 | 128.1 | 1645.3 | 1083.3 |
|  | Median (IQR) | 39051.2 | (25955.9-59070.5) | 830.5 | (0-17764.0) | 0.0 | (0-0) | 42410.4 | (26388.0-69023.1) |
| **SDAC with HSCT** | | | | | | | | | |
| Patient no. | | 361 |  | 305 |  | 223 |  | 361 |  |
| Follow-up time, years | | | | | | | | | |
|  | Mean (SD) | 1.0 | (0.1) | 1.9 | (0.3) | 2.8 | (0.3) | 2.8 | (1.7) |
|  | Median (IQR) | 1.0 | (1.0-1.0) | 2.0 | (2.0-2.0) | 3.0 | (2.7-3.0) | 2.4 | (1.4-4.0) |
| *Medical cost | | | | | | | | | |
|  | Mean (SD) | 86152.6 | 38843.6 | 34864.8 | 37228.7 | 17452.3 | 31482.5 | 134766.2 | 60676.5 |
|  | Median (IQR) | 78592.9 | (58338.6-106518.4) | 20337.8 | (4344.0-54252.7) | 4148.6 | (1350.9-16907.4) | 122227.9 | (89696.4-168415.2) |
| †No. of OPD visits | | | | | | | | | |
|  | Mean (SD) | 34.3 | (15.4) | 43.0 | (25.6) | 35.4 | (24.7) | 114.6 | (91.4) |
|  | Median (IQR) | 33 | (23-43) | 39 | (27-54) | 34 | (19-46) | 102 | (46-154) |
| *Cost of OPD visits | | | | | | | | | |
|  | Mean (SD) | 6648.0 | 6461.5 | 7285.9 | 8916.8 | 4203.7 | 5176.8 | 17984.3 | 16700.9 |
|  | Median (IQR) | 4829 | (2456.4-8120.9) | 4411.0 | (2032.8-8885.1) | 2315.6 | (1135.5-5535.9) | 12387.3 | (6641.9-22229.5) |
| †No. of hospitalization | | | | | | | | | |
|  | Mean (SD) | 5.3 | (1.8) | 2.9 | (1.9) | 2.5 | (2.0) | 8.3 | (4.2) |
|  | Median (IQR) | 5 | (4-6) | 3 | (1-4) | 2 | (1-3) | 8 | (5-10) |
| ‡Length of stay, days | | | | | | | | | |
|  | Mean (SD) | 164.2 | (56.4) | 80.5 | (66.9) | 61.0 | (68.5) | 240.3 | (112.9) |
|  | Median (IQR) | 155 | (123-193) | 66 | (31-122) | 44 | (6-80) | 214 | (161-302) |
| *Cost of hospitalization | | | | | | | | | |
|  | Mean (SD) | 79504.6 | 37269.9 | 27578.9 | 34968.9 | 13248.6 | 29769.4 | 116781.9 | 55011.1 |
|  | Median (IQR) | 71831.8 | (53059.4-99628.3) | 10941.3 | (0-47963.3) | 0 | (0-10555.7) | 103144.5 | (75244.2-150406.7) |
| **SDAC with chemotherapy alone** | | | | | | | | | |
| Patient no. | | 1383 |  | 547 |  | 369 |  | 1383 |  |
| Follow-up time, years | | | | | | | | | |
|  | Mean (SD) | 0.6 | (0.4) | 1.8 | (0.3) | 2.8 | (0.3) | 1.5 | (1.8) |
|  | Median (IQR) | 0.6 | (0.1-1.0) | 2.0 | (1.6-2.0) | 3.0 | (2.8-3.0) | 0.6 | (0.1-2.2) |
| *Medical cost | | | | | | | | | |
|  | Mean (SD) | 40560.2 | 27968.9 | 15730.3 | 25159.0 | 6612.1 | 17392.3 | 49875.8 | 40699.1 |
|  | Median (IQR) | 36199.3 | (19518.2-53801.4) | 3327.0 | (923.5-21094.9) | 1057.5 | (435.7-3035.7) | 41070.4 | (20518.4-67913.6) |
| †No. of OPD visits | | | | | | | | | |
|  | Mean (SD) | 20.3 | (17.9) | 31.4 | (19.2) | 28.7 | (18.3) | 51.2 | (66.7) |
|  | Median (IQR) | 19 | (3-31) | 29 | (18-42) | 27 | (16-39) | 23 | (3-78) |
| *Cost of OPD visits | | | | | | | | | |
|  | Mean (SD) | 2918.8 | 5173.2 | 3910.7 | 8289.6 | 2024.2 | 4179.9 | 5496.8 | 11022.7 |
|  | Median (IQR) | 1266.0 | (398.9-3061.5) | 1546.2 | (681.2-3743.5) | 931.0 | (391.7-1756.0) | 2046.4 | (435.4-5804.4) |
| †No. of hospitalization | | | | | | | | | |
|  | Mean (SD) | 3.6 | (2.4) | 2.6 | (2.1) | 2.9 | (3.4) | 4.5 | (3.9) |
|  | Median (IQR) | 3 | (1-5) | 2 | (1-3) | 2 | (1-4) | 4 | (1-6) |
| ‡Length of stay, days | | | | | | | | | |
|  | Mean (SD) | 96.4 | (64.2) | 55.5 | (51.5) | 46.5 | (46.4) | 113.5 | (88.8) |
|  | Median (IQR) | 97 | (37-137) | 39 | (15-85) | 35 | (8-70) | 104 | (39-156) |
| *Cost of hospitalization | | | | | | | | | |
|  | Mean (SD) | 37641.4 | 26170.5 | 11819.6 | 21641.1 | 4587.9 | 15038.2 | 44378.9 | 35362.4 |
|  | Median (IQR) | 33427.9 | (18370.6-50709.3) | 474.7 | (0-16388.6) | 0 | (0-229.1) | 36968.9 | (18843.8-59946.8) |
| **N-SDAC with HSCT** | | | | | | | | | |
| Patient no. | | 55 |  | 36 |  | 22 |  | 55 |  |
| Follow-up time, years | | | | | | | | | |
|  | Mean (SD) | 0.9 | (0.2) | 1.8 | (0.3) | 2.8 | (0.3) | 2.2 | (1.9) |
|  | Median (IQR) | 1.0 | (0.8-1.0) | 2.0 | (1.4-2.0) | 3.0 | (2.5-3.0) | 1.4 | (0.8-3.4) |
| *Medical cost | | | | | | | | | |
|  | Mean (SD) | 90772.4 | 45080.4 | 32959.9 | 30426.7 | 13836.4 | 22561.5 | 121289.0 | 56427.9 |
|  | Median (IQR) | 79775.9 | (57062.7-121595.2) | 23927.2 | (3891.8-54425.7) | 4594.0 | (1710.9-14858.3 | 120396.4 | (77216.7-152644.4) |
| †No. of OPD visits | | | | | | | | | |
|  | Mean (SD) | 37.8 | (19.1) | 33.6 | (19.0) | 30.1 | (17.8) | 86.8 | (68.3) |
|  | Median (IQR) | 35 | (27-47) | 33 | (19-48) | 29 | (20-33) | 72 | (32-128) |
| *Cost of OPD visits | | | | | | | | | |
|  | Mean (SD) | 10667.8 | 9874.4 | 7226.0 | 8469.7 | 3162.1 | 2739.3 | 18079.7 | 17643.3 |
|  | Median (IQR) | 7633.3 | (4276.8-12962.2) | 3322.7 | (1533.9-9851.1) | 2014.4 | (1151.5-4989.2) | 12113.0 | (7855.9-16562.2) |
| †No. of hospitalization | | | | | | | | | |
|  | Mean (SD) | 4.8 | (2.2) | 2.6 | (1.7) | 2.1 | (1.4) | 6.7 | (4.3) |
|  | Median (IQR) | 4 | (3-6) | 2 | (1-3) | 1 | (1-3) | 5 | (4-9) |
| ‡Length of stay, days | | | | | | | | | |
|  | Mean (SD) | 133.8 | (66.9) | 73.2 | (71.2) | 55.4 | (59.4) | 185.1 | (110.6) |
|  | Median (IQR) | 133 | (96-155) | 46 | (34-96) | 29 | (2-103) | 150 | (111-237) |
| *Cost of hospitalization | | | | | | | | | |
|  | Mean (SD) | 80104.6 | 43225.8 | 25733.8 | 28255.4 | 10674.2 | 22677.4 | 103209.2 | 52624.5 |
|  | Median (IQR) | 70820.1 | (5087.9-108881.8) | 18456.0 | (0-41003.1) | 40.3 | (0-7381.3) | 93439.3 | (65536.1-130228.8) |
| **N-SDAC with chemotherapy alone** | | | | | | | | | |
| Patient no. | | 218 |  | 80 |  | 58 |  | 218 |  |
| Follow-up time, years | | | | | | | | | |
|  | Mean (SD) | 0.6 | (0.4) | 1.8 | (0.3) | 2.9 | (0.3) | 1.4 | (1.8) |
|  | Median (IQR) | 0.5 | (0.2-1.0) | 2.0 | (1.9-2.0) | 3.0 | (2.9-3.0) | 0.5 | (0.2-2.3) |
| *Medical cost | | | | | | | | | |
|  | Mean (SD) | 41495.3 | 27941.9 | 18513.2 | 26132.4 | 8695.9 | 13723.0 | 51453.6 | 43092.5 |
|  | Median (IQR) | 36249.6 | (22150.2-58232.1) | 5375.6 | (1852.9-28849.3) | 2264.1 | (665.4-11240.8) | 40287.7 | (22608.5-66753.8) |
| †No. of OPD visits | | | | | | | | | |
|  | Mean (SD) | 23.5 | (22.3) | 32.9 | (19.1) | 25.0 | (15.7) | 51.1 | (61.3) |
|  | Median (IQR) | 19 | (4-36) | 29 | (20-45) | 22 | (12-34) | 21 | (4-85) |
| *Cost of OPD visits | | | | | | | | | |
|  | Mean (SD) | 6441.5 | 12157.5 | 10281.2 | 19732.8 | 5338.2 | 10305.3 | 12295.7 | 29882.3 |
|  | Median (IQR) | 2386.3 | (760.4-6017.5) | 2652.5 | (1274.3-7036.4) | 1700.8 | (520.7-4670.0) | 3456.4 | (670.4-10955.7) |
| †No. of hospitalization | | | | | | | | | |
|  | Mean (SD) | 3.4 | (2.7) | 2.3 | (1.5) | 1.8 | (1.3) | 4.1 | (3.5) |
|  | Median (IQR) | 3 | (1-4) | 2 | (1-3) | 1 | (1-3) | 3 | (1-5) |
| ‡Length of stay, days | | | | | | | | | |
|  | Mean (SD) | 82.3 | (57.5) | 42.7 | (44.2) | 27.1 | (38.5) | 93.4 | (71.3) |
|  | Median (IQR) | 78 | (33-115) | 28 | (10-59) | 10 | (3-48) | 83 | (39-125) |
| *Cost of hospitalization | | | | | | | | | |
|  | Mean (SD) | 35053.8 | 25699.7 | 8232.0 | 17230.9 | 3357.7 | 8746.2 | 39157.9 | 30245.9 |
|  | Median (IQR) | 29516.1 | (16492.6-49379.0) | 70.5 | (0-6767.8) | 0 | (0-600.2) | 30983.9 | (19475.2-53485.4) |

HDAC: high dose cytarabine, SDAC: standard dose cytarabine, N-SDAC: Non-standard dose.

*total costs were divided by the number of patients who survived the follow up period.

†numbers of outpatient department visit/ hospitalization were divided by number of patients who used healthcare system.

‡total length of stays were divided by number of patients who were hospitalized.
